# Maternal Migration Background and Mortality Among Infants Born Extremely Preterm

**DOI:** 10.1001/jamanetworkopen.2023.47444

**Published:** 2023-12-13

**Authors:** Joaquim Vidiella-Martin, Jasper V. Been

**Affiliations:** 1 Nuffield Department of Primary Care Health Sciences, University of Oxford, Oxford, United Kingdom; 2 Erasmus School of Economics, Tinbergen Institute and Erasmus Centre for Health Economics Rotterdam (EsCHER), Erasmus University Rotterdam, Rotterdam, the Netherlands; 3 Division of Neonatology, Department of Neonatal and Paediatric Intensive Care, Erasmus MC Sophia Children’s Hospital, University Medical Centre Rotterdam, Rotterdam, the Netherlands; 4 Department of Obstetrics and Gynaecology, Erasmus MC Sophia Children’s Hospital, University Medical Centre Rotterdam, Rotterdam, the Netherlands; 5 Department of Public Health, Erasmus MC, University Medical Centre Rotterdam, Rotterdam, the Netherlands

## Abstract

**Importance:**

Extremely preterm infants require care provided in neonatal intensive care units (NICUs) to survive. In the Netherlands, a decision is made regarding active treatment between 24 weeks 0 days and 25 weeks 6 days after consultation with the parents.

**Objective:**

To investigate the association between maternal migration background and admissions to NICUs and mortality within the first year among extremely preterm infants.

**Design, Setting, and Participants:**

This cross-sectional study linked data of registered births in the Netherlands with household-level income tax records and municipality and mortality registers. Eligible participants were households with live births at 24 weeks 0 days to 25 weeks 6 days gestation between January 1, 2010, and December 31, 2017. Data linkage and analysis was performed from March 1, 2020, to June 30, 2023.

**Exposure:**

Maternal migration background, defined as no migration background vs first- or second-generation migrant mother.

**Main Outcomes and Measures:**

Admissions to NICUs and mortality within the first week, month, and year of life. Logistic regressions were estimated adjusted for year of birth, maternal age, parity, household income, sex, gestational age, multiple births, and small for gestational age. NICU-specific fixed effects were also included.

**Results:**

Among 1405 live births (768 male [54.7%], 546 [38.9%] with maternal migration background), 1243 (88.5%) were admitted to the NICU; 490 of 546 infants (89.7%) born to mothers with a migration background vs 753 of 859 infants (87.7%) born to mothers with no migration background were admitted to NICU (fully adjusted RR, 1.03; 95% CI, 0.99-1.08). A total of 652 live-born infants (46.4%) died within the first year of life. In the fully adjusted model, infants born to mothers with a migration background had lower risk of mortality within the first week (RR, 0.81; 95% CI, 0.66-0.99), month (RR, 0.84; 95% CI, 0.72-0.97), and year of life (RR, 0.85; 95% CI, 0.75-0.96) compared with infants born to mothers with no migration background.

**Conclusions:**

In this nationally representative cross-sectional study, infants born to mothers with a migration background at 24 weeks 0 days to 25 weeks 6 days of gestation in the Netherlands had lower risk of mortality within the first year of life than those born to mothers with no migration background, a result that was unlikely to be explained by mothers from different migration backgrounds attending different NICUs or differential preferences for active obstetric management across migration backgrounds. Further research is needed to understand the underlying mechanisms driving these disparities, including parental preferences for active care of extremely preterm infants.

## Key Points

**Question:**

Is maternal migration background associated with neonatal intensive care unit (NICU) admission and survival?

**Findings:**

In this cross-sectional study of 1405 live births in the Netherlands (2010-2017), infants born to migrant mothers between 24 weeks 0 days and 25 weeks 6 days of gestation had lower risk of mortality within the first year of life than infants born to mothers with no migration background. This was unlikely to be explained by differences in admission, in care across NICUs, or in preferences for active obstetric management across migration backgrounds.

**Meaning:**

These results suggest that maternal migration background is associated with increased survival of extremely preterm infants admitted to Dutch NICUs, and further research is needed to understand the underlying factors.

## Teaser

This cross-sectional study of live births in the Netherlands examines the associations between migration background and infant mortality.

## Introduction

Early childhood experiences are often seen as the root of health inequalities.^1,2,3,4^ Therefore, several international organizations have emphasized the need to reduce disparities in birth outcomes between social groups, which are present in developed and developing countries alike.^5,6,7^

Different factors may be behind these disparities, including uneven access to health care, differential health behaviors, or a different provision of health care across patients.^8,9^ Several studies have shown that patients from ethnic minority populations are less likely to receive the best treatments, independent of clinical appropriateness.^10^

Neonatal intensive care units (NICUs) play a pivotal role in ensuring the survival of infants born at the limit of viability. These infants would not survive the early neonatal period in the absence of NICU treatment. Multiple studies have established that many NICU deaths occur in the context of withholding or withdrawing life-sustaining therapies.^11,12^

In the Netherlands, the approach to care at the edge of perinatal viability is characterized by a relatively restrictive threshold of offering active care.^13^ These guidelines are set nationally and establish the same treatment limit in all NICUs in the country. Since 2010, active care is offered to infants born at 24 weeks 0 days gestation and older, with a gray zone spanning 24 and 25 completed weeks.^13^ For infants born in the gray zone, a decision on whether active treatment will be initiated is made before delivery in close communication with parents and following intensive counseling. Around 90% of parents choose active care (instead of palliative care).^14,15^ Neonatal intensive care is included in basic health insurance and provided in 9 tertiary units across the country, thus ensuring equal access to care. Because of this, the study of inequalities within NICUs in the Netherlands can help us learn about the differential care received across sociodemographic groups.

Previous studies have documented large inequalities in perinatal outcomes in the Netherlands across socioeconomic status,^16^ region,^17,18,19^ and ethnicity.^20^ These studies show that adverse perinatal outcomes are more common among socioeconomically deprived households and infants whose mothers come from former Dutch colonies like Suriname or Indonesia. Somewhat surprisingly, a 2021 study^21^ showed that income-related disparities tended to be reversed (with the point estimates favoring the poor, although not statistically significant) among infants born before 26 weeks of gestation (ie, the gray zone).

Outside the Dutch context, the study of the link between ethnicity and outcomes in NICUs has often found that infants belonging to minoritized racial and ethnic groups, especially African American infants, tend to be disadvantaged in the care they receive in NICUs.^22,23^ These differences can partially be explained by the variation of care both across and within NICUs.^24,25,26,27,28^ Recent articles have shown that (at least in the US), immigrant mothers may experience both better and worse perinatal outcomes than nonimmigrant mothers.^29,30^ Some of the observed advantages have been attributed to improved maternal health conditions (the so-called “healthy immigrant paradox”), although further investigation is necessary to comprehensively understand the underlying factors driving this phenomenon.^31^ Because the organization and delivery of health care may not be generalizable from the US to many other countries, the conclusions of these studies do not necessarily apply elsewhere.

In this study, we sought to determine the association between maternal migration background and NICU admission and mortality during the first year among infants born between 24 weeks 0 days and 25 weeks 6 days gestation range in the Netherlands, whose admission was preceded by intensive parental counseling and a joined decision between clinicians and parents whether or not to opt for active care. We estimated this association before and after adjusting for potential confounding factors, such as maternal risk factors and clinical characteristics at birth. As a secondary objective, we evaluated the extent to which inequalities can be explained by unit fixed effects, which capture whether inequalities arise across or within units.

## Methods

We used anonymized data from all registered births in the Netherlands between 2010 and 2017, individually linked to demographic characteristics, mortality, and household-level tax records. This report followed the Strengthening the Reporting of Observational Studies in Epidemiology (STROBE) reporting guideline. Data linkage and analysis was performed from March 1, 2020, to June 30, 2023. Informed consent is not required in the Netherlands when nationwide anonymized data are used. Statistics Netherlands and Perined approved the scope of the research and ensured that no personal information was disclosed.

### Data Sources

Data on all live births from January 1, 2010, until December 31, 2017, between 24 weeks 0 days to 25 weeks 6 days inclusive were obtained from a linked data set of national administrative data accessed remotely via Statistics Netherlands. Stillbirths were not included. The Dutch perinatal registry (Perined) combines medical registries from 4 professional groups that provide perinatal care: general practitioners, midwives, obstetricians, and neonatologists and pediatricians. It contains information on maternal characteristics, pregnancy, delivery, and neonatal outcomes for over 97% of all births in the Netherlands. Annual household income at the individual level was obtained from the Dutch taxation registry. Data on migration background and nationality were obtained from Statistics Netherlands. A detailed description of the linkage procedures among different administrative data sets is provided by Statistics Netherlands.^32^

### Exposure

Maternal migration background was the main exposure variable. Mothers were classified into 1 of 3 categories: no migration background (ie, the mother and both her parents were born in the Netherlands), first-generation migrant, and second-generation migrant. To increase statistical power for our main analyses, we dichotomized migration background into mothers with no migration background and mothers who were first- or second-generation migrants.

### Outcomes

Two sets of outcomes were considered in our study: admissions to NICUs and mortality within the first year of life. The latter was split into mortality within the first week, month, and year of life. Perined provided admissions to NICU and mortality within a week and a month of life. Mortality up to a year of life was provided by the death registry in Statistics Netherlands.

### Potential Confounders

Maternal characteristics included as potential confounders in the analyses were maternal age at delivery (continuous, in years), parity (primiparous vs multiparous), and disposable household income (net of contributions and taxes, such as social security contributions, health insurance, and taxes, and adjusted for household size), categorized into quintiles based on the distribution of the main sample before imputation to account for potential nonmonotonicity of income and survival.^2^ To account for baseline variation in mortality risk of newborns, we included a vector of variables capturing health at birth, before NICU admission. These were sex, gestational age (continuous, given the relationship between gestational age and mortality, [eFigure 1 in Supplement 1]), multiple birth (singleton vs multiple birth), and small for gestational age (SGA) (ie, having a birth weight centile [adjusted for gestational age and sex] below 10 according to the Hoftiezer national reference curves, which are representative of the Dutch population [including migrants]).^33^ To account for year-to-year variation in perinatal mortality, year of birth was also added as a categorical covariate in the model. Selection of potential confounders was based on data availability and recent other work with Perined data.^18,21^

### Missing Data

We had no information on birth outcomes for 18 infants (1.2%) and no information on maternal migration background for 140 live births (9.0%) between 24 weeks 0 days and 25 weeks 6 days registered with Perined. These were excluded from the main sample (eFigure 2 in Supplement 1). There was no information on disposable household income for 15.7% of the sample. For those observations, we imputed the average disposable household income of mothers living in the same postal code area at the time of conception (proxied by subtracting gestational age from the date of birth).

### Statistical Analysis

To examine the association between migration background and our outcomes of interest, 3 logistic regression models were estimated. In the unadjusted model, the outcomes were modeled as a function of migration background and year of birth. In the adjusted model, maternal age at delivery, parity, household income, sex, gestational age, multiple birth, and SGA were added as potential confounders. Finally, in model 3, NICU fixed effects were included in the regression to examine if the estimated results were driven by differences across (eg, different migration backgrounds attending different NICUs, variation in care provided to migrants across different NICUs) or within units (ie, differences remain after accounting for the potentially different distribution of migration backgrounds across units).

Statistical significance was set at *P* < .05 and was evaluated using 2-sided tests. When the incidence of an outcome of interest is common in the study population (above 10%), the adjusted odds ratio derived from the logistic regression can no longer approximate the risk ratio.^34^ Therefore, we report relative risk ratios (RR) and the corresponding 95% CIs. All analyses were performed using Stata version 16 (StataCorp).

To evaluate robustness of our results to the exposure definition (migration background), we performed 2 sensitivity analyses. First, we re-estimated our baseline results separating first- and second-generation migration mothers into 2 different categories. Second, we used information provided by Statistics Netherlands to explore an alternative classification of maternal migration background based on nationality: Dutch, European migrant (including the EU and other non-EU European countries such as Russia, Belarus, Moldova, or Iceland, but not including Turkey), and non-European migrant. This classification follows the latest guidelines by Statistics Netherlands.^35^

We also explored the role of type of delivery. That is, a prenatal decision to prefer comfort care rather than active treatment implies that an instrumental or cesarean delivery will not be performed for fetal concerns. To account for potential bias resulting from any differential distribution of such preferences across migration backgrounds we restricted this sensitivity analysis to spontaneous noninstrumental vaginal deliveries.

Our main analysis of mortality included infants admitted and not admitted to NICUs. We also recalculated our mortality models including only infants that were admitted to NICUs to estimate inequalities after admission to NICU.

We furthermore recalculated our results using a sample of infants born between 26 weeks 0 days and 27 weeks 6 days of gestation. These infants are outside the Dutch gray zone, and active care is in principle provided by default to those infants with a few exceptions (eg, babies with very severe intrauterine growth retardation or those with chromosomal or severe congenital malformations). As such in this gestational age range parental preferences are much less influential in the decision to provide active care at birth. Comparison between the estimates of our primary analyses and the older gestational age sample results can provide insight into any differences in parental preferences for active care management across migration backgrounds.

To ensure that the results were not sensitive to the modeling of household income, 3 other sensitivity analyses were performed. First, we excluded infants for whom we imputed household income and reestimated our results without these observations to account for the downward bias in variability of income arising from mean imputation. Second, we included household income rank as a continuous covariate (ranging from 1 to 100) instead of splitting it into 5 quintiles. Third, we excluded income as a covariate altogether.

## Results

This study included 1405 livebirths (768 male [54.7%], 546 [38.9%] with mothers with migration backgrounds) between 24 weeks 0 days to 25 weeks 6 days of gestation range with complete information on birth outcomes and maternal migration background (Table 1). Among mothers with migration background, 340 of 546 (62.3%) were first-generation migrants and 206 of 546 (37.7%) were second-generation migrants. Mothers with a migration background were more likely to belong to the bottom 2 income quintiles. Infants with no information on birth outcomes (18 infants) or migration background (140 infants) were excluded from the analysis (eFigure 2 in Supplement 1). Infants for whom we had no information on maternal migration background or nationality were more likely to be born before 25 weeks of gestation and less likely to be admitted to the NICU (eTable 1 in Supplement 1).

**Table 1.** Population Characteristics of Live Births by Maternal Migration Background

Characteristics	Participants, No. (%)		
	All (N = 1405)	No migration background (n = 859)	Migration background (n = 546)^a^
**Maternal characteristics**			
Maternal migration background			
No migration background	859 (61.1)	859 (100.0)	0
First generation	340 (24.2)	0	340 (62.3)
Second generation	206 (14.7)	0	206 (37.7)
Maternal age at birth, mean (SD), y	30.2 (5.3)	30.2 (5.0)	30.2 (5.8)
Maternal age at birth, y			
<25	199 (14.2)	110 (12.8)	89 (16.3)
25-34	909 (64.7)	577 (67.2)	332 (60.8)
35-39	240 (17.1)	147 (17.1)	93 (17.0)
>39	57 (4.1)	25 (2.9)	32 (5.9)
Income rank (1-100), mean (SD)^b^	49.1 (29.2)	56.5 (27.4)	37.4 (28.1)
Q1 (lowest)	285 (20.3)	102 (11.9)	183 (33.5)
Q2	282 (20.1)	150 (17.5)	132 (24.2)
Q3	276 (19.6)	179 (20.8)	97 (17.8)
Q4	300 (21.4)	221 (25.7)	79 (14.5)
Q5 (highest)	262 (18.6)	207 (24.1)	55 (10.1)
**Infant characteristics**			
Sex			
Female	637 (45.3)	392 (45.6)	245 (44.9)
Male	768 (54.7)	467 (54.4)	301 (55.1)
Gestational age, wk			
24 + 0 d to 24 + 6 d	646 (46.0)	385 (44.8)	261 (47.8)
25 + 0 d to 25 + 6 d	759 (54.0)	474 (55.2)	285 (52.2)
BWC, mean (SD)	45.7 (30.2)	47.3 (30.7)	43.2 (29.3)
<10	222 (15.8)	127 (14.8)	95 (17.4)
≥10	1183 (84.2)	732 (85.2)	451 (82.6)
Multiple birth			
Singleton	1181 (84.1)	707 (82.3)	474 (86.8)
Multiple birth	224 (15.9)	152 (17.7)	72 (13.2)
Labor			
Spontaneous	1161 (82.6)	724 (84.3)	437 (80.0)
Induced or cesarean delivery	206 (14.7)	117 (13.6)	89 (16.3)
Unclassified	38 (2.7)	18 (2.1)	20 (3.7)
Delivery			
Vaginal	1026 (73.0)	654 (76.1)	372 (68.1)
Cesarean	341 (24.3)	187 (21.8)	154 (28.2)
Unclassified	38 (2.7)	18 (2.1)	20 (3.7)
Admissions to NICU	1243 (88.5)	753 (87.7)	490 (89.7)
Mortality			
First week	361 (25.7)	242 (28.2)	119 (21.8)
First month	544 (38.7)	354 (41.2)	190 (34.8)
First year	652 (46.4)	421 (49.0)	231 (42.3)

Abbreviations: BWC, birth weight centile; NICU, neonatal intensive care unit; Q, quintile.

^a^ Migration background included first- and second-generation migrants.

^b^ Income quintiles calculated based on the distribution of the main sample prior to data imputation.

A total of 1243 infants (88.5%) in our sample were admitted to a NICU. Approximately one-quarter of infants in the sample died within the first week of life (361 infants). This number increased to 544 (38.7%) within the first month of life, and 652 (46.4%) within the first year of life.

### NICU Admission by Migration Background

Among infants whose mothers had a migration background, 490 (89.7%) were admitted to a NICU, compared with 753 infants (87.7%) whose mothers had no migration background (Table 1). In the fully adjusted model, maternal migration background was not significantly associated with admission to NICU (RR, 1.03; 95% CI, 0.99-1.08).

### Mortality by Migration Background

Next, we explored the association between migration background and mortality. Mortality was less common among infants whose mothers had a migration background within the first week of life (119 infants [21.8%] vs 242 [28.2%] for those with mothers without a migration background), the first month (190 infants [34.8%] vs 354 [41.2%]), and the first year (231 [42.3%] vs 421 [49.0%]).

Before adjustment, infants whose mothers had a migration background were less likely to die within the first week of life relative to those whose mothers had no migration background (RR, 0.78; 95% CI, 0.64-0.95) (Table 2). Adjustment for confounding factors led to similar estimates (RR, 0.74; 95% CI, 0.61-0.90). Including NICU fixed effects slightly attenuated estimates (RR, 0.81; 95% CI, 0.66-0.99).

**Table 2.** Association Between Migration Background and NICU Admissions and Mortality

Characteristic	Model 1, RR (95% CI)^a^	Model 2, RR (95% CI)^b^	Model 3, RR (95% CI)^c^
No migration background	1 [Reference]	1 [Reference]	1 [Reference]
NICU admissions (n = 1405)	1.02 (0.98-1.06)	1.04 (1.00-1.08)	1.03 (0.99-1.08)
First-week mortality (n = 1405)	0.78 (0.64-0.95)	0.74 (0.61-0.90)	0.81 (0.66-0.99)
First-month mortality (n = 1405)	0.84 (0.73-0.97)	0.80 (0.70-0.93)	0.84 (0.72-0.97)
First-year mortality (n = 1405)	0.86 (0.76-0.97)	0.81 (0.72-0.92)	0.85 (0.75-0.96)

Abbreviations: NICU, neonatal intensive care unit; RR, risk ratio.

^a^ Unadjusted model including year fixed effects.

^b^ Adjusted for potential confounders, including sex, gestational age (continuous), multiple births, small for gestational age, parity, maternal age at birth (continuous), and household income quintile (categorical).

^c^ Adjusted for potential confounders and including NICU-specific fixed effects.

This pattern persisted when we studied mortality within the first month and the first year of life. In the unadjusted model, the RRs were closer to 1, but still statistically significant (age 1 month: RR, 0.84; 95% CI, 0.73-0.97; age 1 year: RR, 0.86; 95% CI, 0.76-0.97). In the fully adjusted models, the RRs after a month and year of life were similar to those after a week of life (age 1 month: RR, 0.84; 95% CI, 0.72-0.97; age 1 year: RR, 0.85; 95% CI, 0.75-0.96).

### Sensitivity Analyses

Splitting infants whose mothers had a migration background into first generation and second generation did not significantly alter our results (eTable 2 in Supplement 1). Infants in both of these groups were less likely to die within the first year of life.

Using the information on mothers’ nationality instead of migration generation (eTable 3 in Supplement 1) pointed toward those from non-European ascent driving the disparities. Infants whose mothers had European migration background did not show a statistically different rate of admission to NICU relative to infants with Dutch mothers (fully adjusted RR, 0.95; 95% CI, 0.87-1.04) (eTable 4 in Supplement 1). Conversely, infants whose mothers had a non-European migration background were more likely to be admitted to NICU (fully adjusted RR, 1.06; 95% CI, 1.01-1.10). Mortality after a year of life was lower, but not statistically significant among those with European migration background (fully adjusted RR, 0.92; 95% CI, 0.74-1.14), while infants with non-European migrant background were less likely to die after a month and a year of life (fully adjusted RR, 0.83; 95% CI, 0.73-0.95). After restricting the sample to infants born via a spontaneous vaginal delivery, having a mother with first- or second-generation migration background was still associated with lower risk of death (age 1 years: fully adjusted RR, 0.85; 95% CI, 0.73-0.98) (eTable 5 in Supplement 1). After including only children admitted to NICU in the mortality analysis, having a first- or second-generation maternal migration background was still negatively associated with mortality within the first week, month, and year of life (age 1 year: fully adjusted RR, 0.83; 95% CI, 0.72-0.96) (eTable 6 in Supplement 1).

The characteristics of children born between 26 weeks 0 days and 27 weeks 6 days of gestation are reported in eTable 7 in Supplement 1. Among these, the results for maternal migration background were still negative, but were no longer significantly associated with mortality within the first week, month, and year of life (age 1 year: fully adjusted RR, 0.87; 95% CI, 0.72-1.05) (eTable 8 in Supplement 1).

Excluding individuals with imputed household income quintile led to virtually identical estimates, with slightly lower precision arising from a smaller sample (eTable 9 in Supplement 1). Including household income as a continuous covariate or excluding income as a covariate did not alter the estimates (eTables 10 and 11 in Supplement 1).

## Discussion

In this large national study, we investigated disparities in NICU admission and survival rates among extremely preterm infants born at 24 or 25 weeks of gestation in the Netherlands, focusing on maternal migration background. Somewhat unexpectedly, we found that infants with mothers from a first-generation or second-generation migrant background were more likely to survive up to 1 year of life than those with mothers of a nonmigrant background. This finding was robust in sensitivity analyses and appears to be driven primarily by non-European migrants.

Our study accounted for differential distribution of migration backgrounds and/or differential care provided across Dutch NICUs according to varying migration backgrounds, which could potentially influence survival outcomes.^24,25,26^ While we were unable to assess antenatal counseling and perinatal care variations within individual units, our finding that the inclusion of unit fixed effects did not alter the estimated differences across migration backgrounds suggests that disparities in survival are unlikely to reflect differences in the provision of care across NICUs across the country, unlike in other settings like the US.^27,28^

These results contrast with previous research showing that infants who belong to an ethnic minority group tend to have worse outcomes throughout their lifespan,^36^ although this result is conflicting when evaluating neonatal outcomes. While several studies have documented worse outcomes for infants belonging ethnic minority groups,^37,38,39,40,41^ others have found that Black infants born extremely preterm were less likely to die than their White counterparts,^42^ or found no differences regardless of ethnicity.^43^ These findings highlight the challenge of international comparisons when it comes to nativity, ethnicity, and race research.^44^ It is important to note that migration background and nationality, the markers used in our study, are imperfect representations of ethnicity, which in itself is a complex concept including cultural, racial, and geographical aspects.

Furthermore, restricting the analysis to spontaneous vaginal deliveries did not substantially affect the study’s conclusion. This result suggests that differential preference for active obstetric management at 24 weeks 0 days to 25 weeks 6 days is unlikely to be the driving factor behind our results.

One possible explanation for our findings is that mothers with a migration background may be more likely to opt for active management at birth or less likely to accept withdrawal. This preference could result in more NICU admissions and a lower likelihood of discontinuing intensive care for their 24- or 25-week-old infants during the initial weeks of treatment. Recent evidence has demonstrated that parental preferences for active care management of preterm infants may not be based on objective health measures at birth.^45^ Additionally, other studies have shown that parents consider factors beyond probabilistic facts about expected outcomes when making end-of-life decisions for their children, including religious faith, which may partially account for the variation across migration backgrounds in periviable decision-making.^46,47,48,49,50,51^ Among infants born at 26 weeks 0 days to 27 weeks 6 days, who are almost always provided active care and among whom parental preferences may play a less important role in this decision, we found no significant differences despite larger sample sizes.

Furthermore, underlying differences in the etiology of preterm birth across ethnicities may partially explain the variations in mortality rates observed across different migration backgrounds. A 2022 meta-study^52^ demonstrated that babies born to Black women were at an increased risk of preterm birth.

### Limitations

Several limitations to our study should be acknowledged. First, due to the rarity of extremely preterm births before week 26 of gestation, our sample size was relatively small. Additionally, maternal migration background and nationality may not fully capture the diversity encompassed by ethnicity. It is important to consider the limitations of these markers in interpreting our results.

We also acknowledge the exclusion of infants for whom we had no information on maternal migration background or nationality, which may have introduced some bias in our estimates. Our study may be subject to bias arising from selection into survival at birth. Although we attempted a sensitivity analysis to address this by focusing only on infants born following noninstrumental vaginal delivery, the lack of detailed data on stillborn infants born extremely preterm limited our ability to fully account for this potential bias.

Another limitation is that we lacked reliable information on causes of death or common complications of preterm birth. We also had no information on end-of-life decisions, which hinders our comprehensive understanding of the factors driving the patterns observed in our study. Lastly, unobserved lifestyle factors such as maternal smoking during pregnancy may be a source of residual confounding in our study results.

## Conclusions

Our study reveals that infants born at 24 weeks 0 days to 25 weeks 6 days in the Netherlands have a higher chance of survival up to 1 year of life if their mothers have a migration background. This finding contradicts the typical trend of nonmigrant children having better outcomes in early life. This research emphasizes the urgency of conducting additional studies to investigate the underlying factors behind this, specifically regarding the varying values and preferences for intensive care at birth and on withholding and withdrawing intensive care among different ethnic and migrant groups.

## Notes

Supplement 1. **eTable 1.** Population Characteristics of the Livebirths Without Information on Maternal Migration Background **eTable 2.** Association Between Migration Background and NICU Admissions and Mortality (Within the First Week, Month, and Year of Life), Separating First-Generation and Second-Generation Migration Background **eTable 3.** Mapping Between Migration Background by Generation and by Region of Origin **eTable 4.** Association Between Maternal Migration Background and NICU Admissions and Mortality (Within the First Week, Month, and Year of Life), Using Nationality to Define Migration Background **eTable 5.** Association Between Maternal Migration Background and NICU Admissions and Mortality (Within the First Week, Month, and year of life), Restricting the Sample to Spontaneous Deliveries **eTable 6.** Association Between Maternal Migration Background and Mortality (Within the First Week, Month, and Year of Life), Restricting the Sample to Livebirths Admitted to NICU **eTable 7.** Population Characteristics of Livebirths Between 26 Weeks 0 Days and 27 Weeks 6 Days Gestation Range by Maternal Migration Background **eTable 8.** Association Between Maternal Migration Background and NICU Admission of Livebirths and Mortality (Within the First Week, Month, and Year of Life), Restricting the Sample to Infants Born Between 26 Weeks 0 Days and 27 Weeks 6 Days Gestation Range **eTable 9.** Association Between Maternal Migration Background and NICU Admissions and Mortality (Within the First Week, Month, and Year of Life), Excluding Infants With Imputed Household Income **eTable 10.** Association Between Maternal Migration Background and NICU Admissions and Mortality (Within the First Week, Month, and Year of Life), Modeling Household Income as a Continuous Covariate Instead of Quintiles **eTable 11.** Association Between Maternal Migration Background and NICU Admissions and Mortality (Within the First Week, Month, and Year of Life), Excluding Income as a Potential Confounder **eFigure 1.** Mean Mortality After a Year of Life by Gestational Age **eFigure 2.** Population Flow Diagram

Supplement 2.
Data Sharing Statement
