# Supplementary material for: Maternal Migration Background and Mortality Among Infants Born Extremely Preterm
Source: JAMA Netw Open. 2023 Dec 13;6(12):e2347444. doi: 10.1001/jamanetworkopen.2023.47444 (PMC10719757; doi:10.1001/jamanetworkopen.2023.47444)
Supplement: Supplement 1. — eTable 1. Population Characteristics of the Livebirths Without Information on Maternal Migration Background eTable 2. Association Between Migration Background and NICU Admissions and Mortality (Within the First Week, Month, and Year of Life), Separating First-Generation and Second-Generation Migration Background eTable 3. Mapping Between Migration Background by Generation and by Region of Origin eTable 4. Association Between Maternal Migration Background and NICU Admissions and Mortality (Within the First Week, Month, and Year of Life), Using Nationality to Define Migration Background eTable 5. Association Between Maternal Migration Background and NICU Admissions and Mortality (Within the First Week, Month, and year of life), Restricting the Sample to Spontaneous Deliveries eTable 6. Association Between Maternal Migration Background and Mortality (Within the First Week, Month, and Year of Life), Restricting the Sample to Livebirths Admitted to NICU eTable 7. Population Characteristics of Livebirths Between 26 Weeks 0 Days and 27 Weeks 6 Days Gestation Range by Maternal Migration Background eTable 8. Association Between Maternal Migration Background and NICU Admission of Livebirths and Mortality (Within the First Week, Month, and Year of Life), Restricting the Sample to Infants Born Between 26 Weeks 0 Days and 27 Weeks 6 Days Gestation Range eTable 9. Association Between Maternal Migration Background and NICU Admissions and Mortality (Within the First Week, Month, and Year of Life), Excluding Infants With Imputed Household Income eTable 10. Association Between Maternal Migration Background and NICU Admissions and Mortality (Within the First Week, Month, and Year of Life), Modeling Household Income as a Continuous Covariate Instead of Quintiles eTable 11. Association Between Maternal Migration Background and NICU Admissions and Mortality (Within the First Week, Month, and Year of Life), Excluding Income as a Potential Confounder eFigure 1. Mean Mortality After a Year [file jamanetwopen-e2347444-s001.pdf]

Supplemental Online Content

Vidiella-Martin J, Been JV. Maternal migration background and mortality among infants born extremely preterm. *JAMA Netw Open.* 2023;6(12):e2347444.
doi:10.1001/jamanetworkopen.2023.47444

eTable 1. Population Characteristics of the Livebirths Without Information on Maternal Migration Background

eTable 2. Association Between Migration Background and NICU Admissions and Mortality (Within the First Week, Month, and Year of Life), Separating First-Generation and Second-Generation Migration Background

eTable 3. Mapping Between Migration Background by Generation and by Region of Origin

eTable 4. Association Between Maternal Migration Background and NICU Admissions and Mortality (Within the First Week, Month, and Year of Life), Using Nationality to Define Migration Background

eTable 5. Association Between Maternal Migration Background and NICU Admissions and Mortality (Within the First Week, Month, and year of life), restricting the sample to spontaneous deliveries

eTable 6. Association Between Maternal Migration Background and Mortality (Within the First Week, Month, and Year of Life), Restricting the Sample to Livebirths Admitted to NICU

eTable 7. Population Characteristics of Livebirths Between 26 Weeks 0 Days and 27 Weeks 6 Days Gestation Range by Maternal Migration Background

eTable 8. Association Between Maternal Migration Background and NICU Admission of Livebirths and Mortality (Within the First Week, Month, and Year of

© 2023 Vidiella-Martin et al. *JAMA Network Open.*

Life), Restricting the Sample to Infants Born Between 26 Weeks 0 Days and 27 Weeks 6 Days Gestation Range

eTable 9. Association Between Maternal Migration Background and NICU Admissions and Mortality (Within the First Week, Month, and Year of Life), Excluding Infants With Imputed Household Income

eTable 10. Association Between Maternal Migration Background and NICU Admissions and Mortality (Within the First Week, Month, and Year of Life), Modeling Household Income as a Continuous Covariate Instead of Quintiles

eTable 11. Association Between Maternal Migration Background and NICU Admissions and Mortality (Within the First Week, Month, and Year of Life), Excluding Income as a Potential Confounder

eFigure 1. Mean Mortality After a Year of Life by Gestational Age

eFigure 2. Population Flow Diagram

This supplemental material has been provided by the authors to give readers additional information about their work.

© 2023 Vidiella-Martin et al. *JAMA Network Open.*

eTable 1: Population characteristics of the livebirths without information on maternal migration background

	No maternal migration background info (n=140)	Main sample (n=1,405)
**Maternal characteristics**		
Maternal age at birth, mean (SD)	30.2 (5.3)	30.2 (5.3)
<25	13 (9.3)	199 (14.2)
25-34	97 (69.3)	909 (64.7)
>34	30 (18.6)	297 (21.1)
Income rank (1-100), mean (SD)	47 (22.1)	49.1 (29.2)
Q1 (lowest)	17 (12.1)	285 (20.3)
Q2	36 (25.7)	282 (20.1)
Q3	48 (34.3)	276 (19.6)
Q4	28 (20.0)	300 (21.4)
Q5 (highest)	11 (7.9)	262 (18.6)
**Infant characteristics**		
Sex		
Female	52 (37.1)	637 (45.3)
Male	88 (62.9)	768 (54.7)
Gestational age		
24+0 to 24+6 weeks	82 (58.6)	646 (46.0)
25+0 to 25+6 weeks	58 (41.4)	759 (54.0)
Birth weight centile, mean (SD)	45 (33.2)	45.7 (30.2)
BWC <10	30 (21.4)	222 (15.8)
BWC ≥10	110 (78.6)	1,183 (84.2)
Multiple birth		
Singleton	114 (81.4)	1181 (84.1)
Multiple birth	26 (18.6)	224 (15.9)
Labor		
Spontaneous	93 (66.4)	1,161 (82.6)
Induced/C-section	27 (19.2)	206 (14.7)
Unclassified	10 (7.1)	38 (2.7)
Delivery		
Vaginal	106 (75.7)	1,026 (73)
C-section	14 (10.0)	341 (24.3)
Unclassified	20 (14.3)	38 (2.7)
Admissions to NICU	70 (50.0)	1,243 (88.5)
Mortality		
First week	69 (49.3)	361 (25.7)
First month	80 (57.1)	544 (38.7)
First year	N/A	652 (46.4)

© 2023 Vidiella-Martin et al. *JAMA Network Open.*

Data are presented as numbers (percentages) unless otherwise indicated.
BWC = Birth weight centile.
Income quintiles calculated based on the distribution of the main sample prior to data imputation
Age categories 35-39 and >39 pooled together to comply with the regulations by Statistics Netherlands.
First-year mortality not available for infants without maternal migration background, as they cannot be linked throughout different datasets.

© 2023 Vidiella-Martin et al. *JAMA Network Open.*

eTable 2: Association between maternal migration background and NICU admissions and mortality (within the first week, month, and year of life), separating first-generation and second-generation migration background

	Model 1	Model 2	Model 3
	**NICU admissions (N=1,405)**		
First-generation migrant	1.00 (0.96-1.05)	1.03 (0.98-1.07)	1.02 (0.97-1.07)
Second-generation migrant	1.06 (1.01-1.11)	1.06 (1.01-1.11)	1.05 (1.00-1.11)
	**First-week mortality (N=1,405)**		
First-generation migrant	0.86 (0.69-1.07)	0.77 (0.61-0.97)	0.84 (0.67-1.07)
Second-generation migrant	0.65 (0.48-0.89)	0.69 (0.52-0.93)	0.76 (0.57-1.02)
	**First-month mortality (N=1,405)**		
First-generation migrant	0.93 (0.79-1.08)	0.85 (0.72-1.01)	0.89 (0.75-1.05)
Second-generation migrant	0.71 (0.57-0.89)	0.73 (0.59-0.91)	0.76 (0.61-0.94)
	**First-year mortality (N=1,405)**		
First-generation migrant	0.91 (0.79-1.04)	0.83 (0.72-0.96)	0.87 (0.75-1.00)
Second-generation migrant	0.77 (0.64-0.93)	0.79 (0.67-0.95)	0.83 (0.69-0.98)

Risk ratios (RR) of first-generation migration background and second-generation migration background. Reference category = no migration background. 95% confidence intervals in brackets.
Model 1: unadjusted model including year fixed effects.
Model 2: adjusted for potential confounders - sex, gestational age (continuous), multiple births, small for gestational age, parity, maternal age at birth (continuous), and household income quintile (categorical).
Model 3: adjusted for potential confounders and including neonatal intensive care unit-specific fixed effects.

© 2023 Vidiella-Martin et al. *JAMA Network Open.*

eTable 3: Mapping between maternal migration background by generation and by region of origin

	Dutch	European migrant	Non-European migrant	Total
No migration background	859	0	0	859
First-generation migrant	0	82	23	105
Second-generation migrant	0	258	183	441
Total	859	340	206	1,405

© 2023 Vidiella-Martin et al. *JAMA Network Open.*

eTable 4: Association between maternal migration background and NICU admissions and mortality (within the first week, month, and year of life), using nationality to define migration background

	Model 1	Model 2	Model 3
	**NICU admissions (N=1,405)**		
European migrant	0.94 (0.85-1.03)	0.95 (0.87-1.04)	0.95 (0.87-1.04)
Non-European migrant	1.04 (1.00-1.08)	1.06 (1.02-1.10)	1.06 (1.01-1.10)
	**First-week mortality (N=1,405)**		
European migrant	0.94 (0.67-1.32)	0.89 (0.64-1.25)	0.98 (0.70-1.36)
Non-European migrant	0.74 (0.60-0.92)	0.70 (0.57-0.87)	0.77 (0.62-0.96)
	**First-month mortality (N=1,405)**		
European migrant	1.01 (0.79-1.28)	0.97 (0.77-1.23)	1.02 (0.81-1.29)
Non-European migrant	0.81 (0.69-0.94)	0.76 (0.65-0.89)	0.79 (0.67-0.93)
	**First-year mortality (N=1,405)**		
European migrant	0.90 (0.72-1.13)	0.87 (0.70-1.08)	0.92 (0.74-1.14)
Non-European migrant	0.85 (0.75-0.97)	0.80 (0.70-0.91)	0.83 (0.73-0.95)

Risk ratios (RR) of European migration background or non-European migration background. Reference category = Dutch. 95% confidence intervals in brackets.
Model 1: unadjusted model including year fixed effects.
Model 2: adjusted for potential confounders - sex, gestational age (continuous), multiple births, small for gestational age, parity, maternal age at birth (continuous), and household income quintile (categorical).
Model 3: adjusted for potential confounders and including neonatal intensive care unit-specific fixed effects.

© 2023 Vidiella-Martin et al. *JAMA Network Open.*

eTable 5: Association between maternal migration background and NICU admissions and mortality (within the first week, month, and year of life), restricting the sample to spontaneous vaginal deliveries

	Model 1	Model 2	Model 3
NICU admissions (N=984)	1.01 (0.96-1.06)	1.02 (0.97-1.07)	1.01 (0.96-1.07)
First-week mortality (N=984)	0.78 (0.62-0.97)	0.72 (0.57-0.91)	0.78 (0.62-0.99)
First-month mortality (N=984)	0.85 (0.72-1.00)	0.81 (0.68-0.96)	0.84 (0.71-1.00)
First-year mortality (N=984)	0.85 (0.74-0.98)	0.81 (0.70-0.93)	0.85 (0.73-0.98)

Risk ratios (RR) of first-or-second-generation migration background. Reference category = no migration background. 95% confidence intervals in brackets.
Model 1: unadjusted model including year fixed effects.
Model 2: adjusted for potential confounders - sex, gestational age (continuous), multiple births, small for gestational age, parity, maternal age at birth (continuous), and household income quintile (categorical).
Model 3: adjusted for potential confounders and including neonatal intensive care unit-specific fixed effects.

© 2023 Vidiella-Martin et al. *JAMA Network Open.*

eTable 6: Association between maternal migration background and mortality (within the first week, month, and year of life), restricting the sample to livebirths admitted to NICU

	Model 1	Model 2	Model 3
First-week mortality (N=1,243)	0.74<br>(0.57-0.96)	0.73<br>(0.56-0.96)	0.82<br>(0.63-1.08)
First-month mortality (N=1,243)	0.83<br>(0.70-0.99)	0.80<br>(0.67-0.95)	0.83<br>(0.69-0.99)
First-year mortality (N=1,243)	0.85<br>(0.74-0.98)	0.80<br>(0.69-0.92)	0.83<br>(0.72-0.96)

Risk ratios (RR) of first-or-second-generation migration background. Reference category = no migration background. 95% confidence intervals in brackets.

Model 1: unadjusted model including year fixed effects.

Model 2: adjusted for potential confounders - sex, gestational age (continuous), multiple births, small for gestational age, parity, maternal age at birth (continuous), and household income quintile (categorical).

Model 3: adjusted for potential confounders and including NICU-specific fixed effects.

© 2023 Vidiella-Martin et al. *JAMA Network Open.*

eTable 7: Population characteristics of livebirths between 26 weeks 0 days and 27 weeks 6 days gestation range by maternal migration background

	All (n=2,456)	No migration background (n=1,584)	First-or-second-generation migrant (n=872)
**Maternal characteristics**			
Maternal migration background			
No migration background	1,584 (64.5)	1,584 (100.0)	0 (0.0)
First-generation migrant	545 (22.2)	0 (0.0)	545 (62.5)
Second-generation migrant	327 (13.3)	0 (0.0)	327 (37.5)
Maternal age at birth, mean (SD)	30.3 (5.4)	30.1 (5.1)	30.4 (6.0)
<25	343 (14.0)	198 (12.5)	145 (16.6)
25-34	1,573 (64.0)	1,078 (68.1)	495 (56.8)
35-39	421 (17.1)	248 (15.7)	173 (19.8)
>39	119 (4.8)	60 (3.8)	59 (6.8)
Income rank (1-100), mean (SD)	48.6 (28.5)	54.5 (27.0)	37.9 (28.0)
Q1 (lowest)	512 (20.8)	205 (12.9)	307 (35.2)
Q2	482 (19.6)	293 (18.5)	189 (21.7)
Q3	474 (19.3)	323 (20.4)	151 (17.3)
Q4	505 (20.6)	390 (24.6)	115 (13.2)
Q5 (highest)	483 (19.7)	373 (23.5)	110 (12.6)
**Infant characteristics**			
Sex			
Female	1,098 (44.7)	696 (43.9)	402 (46.1)
Male	1,358 (55.3)	888 (56.1)	470 (53.9)
Gestational age			
24+0 to 24+6 weeks	1,176 (47.9)	750 (47.3)	426 (48.9)
25+0 to 25+6 weeks	1,280 (52.1)	834 (52.7)	446 (51.1)
Birth weight centile, mean (SD)	36.9 (31.0)	38.6 (31.6)	33.9 (29.6)
BWC <10	711 (28.9)	437 (27.6)	274 (31.4)
BWC ≥10	1,745 (71.1)	1147 (72.4)	598 (68.6)
Multiple birth			
Singleton	2,100 (85.5)	1337 (84.4)	763 (87.5)
Multiple birth	356 (14.5)	247 (15.6)	109 (12.5)
Labor			
Spontaneous	1,478 (60.1)	963 (60.8)	515 (59.1)
Induced/C-section	922 (36.7)	581 (36.7)	341 (39.1)
Unclassified	56 (2.3)	40 (2.5)	16 (1.8)
Delivery			
Vaginal	1,077 (43.9)	707 (44.6)	370 (42.4)
C-section	1,323 (53.9)	837 (52.8)	486 (55.7)
Unclassified	56 (2.3)	40 (2.5)	16 (1.8)

© 2023 Vidiella-Martin et al. *JAMA Network Open.*

Admissions to NICU	2,380 (96.9)	1,536 (97)	844 (96.8)
Mortality			
First week	197 (8.0)	137 (8.6)	60 (6.9)
First month	328 (13.4)	219 (13.8)	109 (12.5)
First year	451 (18.4)	299 (18.9)	152 (17.4)

Data are presented as numbers (percentages) unless otherwise indicated.
BWC = Birth weight centile.
Income quintiles calculated based on the distribution of the main sample prior to data imputation

© 2023 Vidiella-Martin et al. *JAMA Network Open.*

eTable 8: Association between maternal migration background and NICU admissions and mortality (within the first week, month, and year of life), restricting the sample to infants born between 26 weeks 0 days and 27 weeks 6 days gestation range

	Model 1	Model 2	Model 3
NICU admissions (N=2,456)	1.00 (0.98-1.01)	1.00 (0.99-1.02)	1.01 (0.99-1.02)
First-week mortality (N=2,456)	0.80 (0.60-1.07)	0.74 (0.55-1.00)	0.74 (0.54-1.01)
First-month mortality (N=2,456) .	0.91 (0.74-1.13)	0.87 (0.70-1.09)	0.83 (0.66-1.05)
First-month mortality (N=2,456)	0.92 (0.77-1.10)	0.89 (0.74-1.06)	0.87 (0.72-1.05)

Risk ratios (RR) of first-or-second-generation migration background. Reference category = no migration background. 95% confidence intervals in brackets.
Model 1: unadjusted model including year fixed effects.
Model 2: adjusted for potential confounders - sex, gestational age (continuous), multiple births, small for gestational age, parity, maternal age at birth (continuous), and household income quintile (categorical).
Model 3: adjusted for potential confounders and including neonatal intensive care unit-specific fixed effects.

© 2023 Vidiella-Martin et al. *JAMA Network Open.*

eTable 9: Association between maternal migration background and NICU admissions and mortality (within the first week, month, and year of life), excluding infants with imputed household income

	Model 1	Model 2	Model 3
NICU admissions (N=1,185)	1.02 (0.98-1.07)	1.05 (1.00-1.09)	1.04 (1.00-1.09)
First-week mortality (N=1,185)	0.77 (0.63-0.96)	0.74 (0.59-0.93)	0.83 (0.66-1.04)
First-month mortality (N=1,185)	0.81 (0.69-0.95)	0.78 (0.66-0.92)	0.81 (0.68-0.96)
First-year mortality (N=1,185)	0.83 (0.73-0.95)	0.79 (0.69-0.91)	0.83 (0.72-0.95)

Risk ratios (RR) of first-or-second-generation migration background. Reference category = no migration background. 95% confidence intervals in brackets.
Model 1: unadjusted model including year fixed effects.
Model 2: adjusted for potential confounders - sex, gestational age (continuous), multiple births, small for gestational age, parity, maternal age at birth (continuous), and household income quintile (categorical).
Model 3: adjusted for potential confounders and including NICU-specific fixed effects.

© 2023 Vidiella-Martin et al. *JAMA Network Open.*

eTable 10: Association between maternal migration background and NICU admissions and mortality (within the first week, month, and year of life), modeling household income rank as a continuous covariate instead of quintiles

	Model 1	Model 2	Model 3
NICU admissions (N=1,405)	1.02 (0.98-1.06)	1.04 (1.00-1.08)	1.03 (0.99-1.07)
First-week mortality (N=1,405)	0.78 (0.64-0.95)	0.75 (0.62-0.91)	0.83 (0.68-1.01)
First-month mortality (N=1,405)	0.84 (0.73-0.97)	0.81 (0.71-0.94)	0.85 (0.73-0.98)
First-year mortality (N=1,405)	0.86 (0.76-0.97)	0.82 (0.73-0.93)	0.86 (0.76-0.97)

Risk ratios (RR) of first-or-second-generation migration background. Reference category = no migration background. 95% confidence intervals in brackets.
Model 1: unadjusted model including year fixed effects.
Model 2: adjusted for potential confounders - sex, gestational age (continuous), multiple births, small for gestational age, parity, maternal age at birth (continuous), and household income rank (continuous).
Model 3: adjusted for potential confounders and including neonatal intensive care unit-specific fixed effects.

© 2023 Vidiella-Martin et al. *JAMA Network Open.*

eTable 11: Association between maternal migration background and NICU admissions and mortality (within the first week, month, and year of life), excluding household income as covariate

	Model 1	Model 2	Model 3
NICU admissions (N=1,405)	1.02 (0.98-1.06)	1.04 (1.00-1.08)	1.03 (0.99-1.07)
First-week mortality (N=1,405)	0.78 (0.64-0.95)	0.73 (0.60-0.88)	0.79 (0.65-0.96)
First-month mortality (N=1,405)	0.84 (0.73-0.97)	0.80 (0.69-0.91)	0.83 (0.72-0.95)
First-year mortality (N=1,405)	0.86 (0.76-0.97)	0.81 (0.72-0.91)	0.84 (0.75-0.95)

Risk ratios (RR) of first-or-second-generation migration background. Reference category = no migration background. 95% confidence intervals in brackets.
Model 1: unadjusted model including year fixed effects.
Model 2: adjusted for potential confounders - sex, gestational age (continuous), multiple births, small for gestational age, parity, and maternal age at birth (continuous).
Model 3: adjusted for potential confounders and including neonatal intensive care unit-specific fixed effects.

© 2023 Vidiella-Martin et al. *JAMA Network Open.*

eFigure 1: Mean mortality after a year of life by gestational age

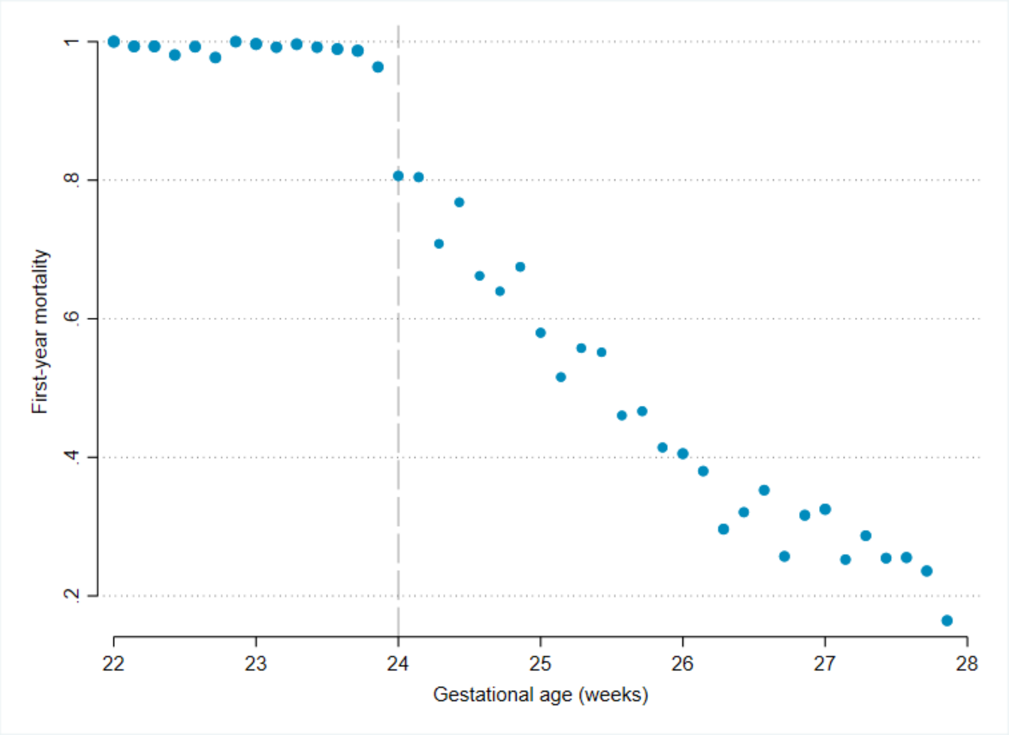

Each dot depicts the mean first-year mortality rate by gestational age (in days), with gestational age ranging from 22 weeks 0 days to 27 weeks 6 days. The vertical dashed line represents the 24 weeks 0 days cut-off for active care management in Dutch NICUs.

© 2023 Vidiella-Martin et al. *JAMA Network Open.*

eFigure 2: Population flow diagram

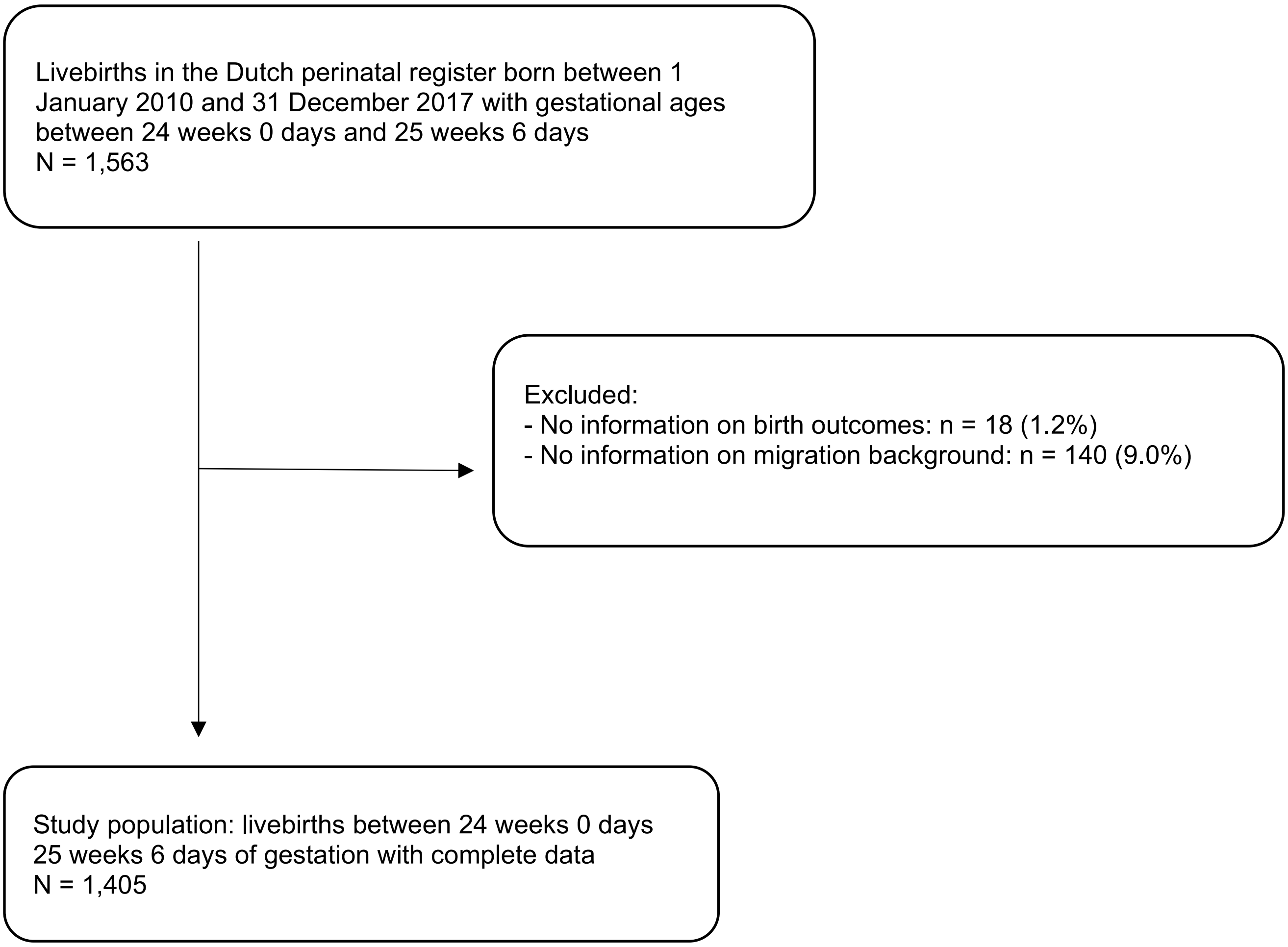

© 2023 Vidiella-Martin et al. *JAMA Network Open.*
