# Supplementary material for: Maternal Migration Background and Mortality Among Infants Born Extremely Preterm
Source: JAMA Netw Open. 2023 Dec 13;6(12):e2347444. doi: 10.1001/jamanetworkopen.2023.47444 (PMC10719757; doi:10.1001/jamanetworkopen.2023.47444)
Supplement: Supplement 2. — Data Sharing Statement [file jamanetwopen-e2347444-s002.pdf]

Data Sharing Statement

Vidiella-Martin. Maternal Migration Background and Mortality Among Infants Born Extremely Preterm. *JAMA Netw Open*. Published December 13, 2023.
doi:10.1001/jamanetworkopen.2023.47444

Data
Data available: No

Additional Information
Explanation for why data not available: The study uses non-public microdata provided by Statistics Netherlands via a Remote Access facility and complies with the respective data agreement. As stipulated in the agreement, Statistics Netherlands pre-viewed the findings before publication to ensure that privacy-sensitive, individual-specific information is not revealed. The data from this study can only be applied through a government data-sharing portal of Statistics Netherlands (https://www.cbs.nl/en-gb/our-services/customised-services-microdata/microdata-conducting-your-own-research). Under certain conditions, this microdata is accessible for statistical and scientific research. For further information: microdata@cbs.nl.
